# Weight stigma, welfare stigma, and political values: evidence from a representative British survey

**DOI:** 10.1016/j.socscimed.2023.116172

**Published:** 2023-08-18

**Authors:** Amanda M Hughes, Daniel McArthur

**Affiliations:** a MRC Integrative Epidemiology Unit, Department of Population Health Sciences, University of Bristol, UK; b Department of Education, University of York, UK

**Keywords:** obesity stigma, weight stigma, stigma, political values, political attitudes, authoritarianism, libertarianism, welfare stigma

## Abstract

Obesity-related stigma is increasingly recognised as a public health issue, with implications for the mental and physical health of people with and without obesity. However, very little is known about what drives inter-individual differences in obesity-stigmatizing views, and how they are distributed in the population. If views about obesity are not independent of a person’s wider beliefs and values, this must be understood so that obesity stigma can be effectively tackled.

In a representative sample of British adults aged 18-97 (N=2186), we explore predictors of weight-stigmatizing attitudes. We consider demographics, socioeconomic position, factors related to one’s own weight and health, and beliefs about the causes and consequences of obesity. We explore the role of core political values which predict views about other stigmatized groups, and views about welfare recipients, who are frequently linked with obesity in public and political discourse. Finally, we assess to what extent demographic differences in weight-stigmatizing attitudes are explained by individual body mass index (BMI), attitudes, and beliefs.

Consistent with previous studies, women were less weight-stigmatizing than men. People in late middle-age were less weight-stigmatizing than younger or older adults. Adjusted for age and gender, an index of weight-stigmatizing views was positively associated with income, and highest in intermediate categories of education and occupational social class. Weight-stigmatizing attitudes were associated with more right-wing values, more authoritarian values, and more stigmatizing views about welfare recipients. Factors including own BMI, beliefs about causes of obesity, welfare-stigmatizing attitudes and authoritarian values contributed to socioeconomic differences.

Weight-stigmatizing attitudes show clear differences between demographic groups, but also vary according to wider social attitudes, beliefs, and a person’s core political values. Efforts to reduce weight stigma, and other kinds of stigma, may be more effective if they recognise these links.

## Background

Obesity is common, affecting 26% of adults and 23% of children in England(1) but it is also highly stigmatized. A visible transgression of a social norm(2), obesity elicits reactions of blame and dislike(3). Weight-related discrimination is widely observed in medical(4,5), educational(6,7) and workplace settings(8,9), which can damage health and social functioning in diverse ways. Experience of weight stigma is associated with impaired mental health(10,11) and worse quality of life(12). Weight stigma by doctors(13) is a recognised barrier to health service use for people with obesity(14). Moreover, evidence suggests that weight stigma can negatively affect eating patterns(15), act as a barrier to physical activity(16,17), and increase weight gain over time(18). Internalized weight stigma, when people come to believe that negative obesity-related stereotypes apply to themselves(19), is linked to disordered eating for overweight (20), normal-weight and underweight individuals(21), and is thus relevant to mental health across the body weight range. Weight stigma is becoming recognised as a public health problem in its own right(22,23), with increasing acknowledgment by researchers that public health initiatives which aim to reduce obesity may contribute to weight stigma(24). There is widespread concern that focus on weight control during COVID-19 lockdowns may have exacerbated these problems(25), contributing to an unprecedented increase in referrals to eating disorder services(26).

Stigmatization can be understood as a process(27).Weight stigma research has largely focused on two elements of the process: on ‘experienced’ weight stigma, or weight-related discrimination(28–30), and ‘internalized’ weight stigma, self-attribution of negative obesity-related stereotypes(31–33). In contrast, research into who holds weight-stigmatizing attitudes, and why, is less extensive. This question has been approached in two ways: by looking at explicit, or expressed, attitudes (13,34–36), and by investigating implicit bias against people with obesity(13,37,38). These studies have often suggested that men are more weight-stigmatizing than women(36,39), but not always(35). US research has found mixed evidence for differences by ethnicity (36,40,41). Findings are inconclusive for age and education(35,36,39,42), and a recent systematic review noted a lack of studies on the role of socioeconomic factors(43). Like other weight stigma research, studies have often used nonrepresentative sample populations and focused on the United States(13,34,36). UK-based work on weight-stigmatizing attitudes has investigated the impact of age, gender, own body mass index (BMI), and exercise frequency(37,39), but not socioeconomic factors or ethnicity.

Perceived causes of stigmatizing characteristics can affect how much they evoke dislike and anger, and whether affected individuals are deemed deserving of help(3). People who see obesity as a matter of personal responsibility have more weight-stigmatizing attitudes(35,36,42), and it has therefore been suggested that stigma could be reduced by educating people about wider drivers of obesity(44). However, intervention studies suggest that providing such information, or evoking empathy, is likely to have limited effects(44–47). This indicates that other influences may be at play, and that weight-stigmatizing attitudes may be rooted in more fundamental beliefs and values. US studies into ideological correlates of weight stigma have reported associations with “just world beliefs” (the belief that people largely get what they deserve in life), and the Protestant work ethic (the belief in the moral value of hard work and self-discipline) (48–50). They also suggest that conservatives may have more weight-stigmatizing attitudes than liberals(50,51).

Results such as these indicate that weight stigma may have deeper origins which are related to a person’s other social and political views. One explanation is that attitudes about obesity are not independent of attitudes about social groups where obesity is, or is perceived to be, common. Among the most consistent findings in social epidemiology is that obesity is associated with socioeconomic disadvantage, at least in higher-income countries(52). It is also more common among people affected by mental illness(53,54) and some ethnic minority groups(55). This raises the possibility that negative views about obesity may partly be driven by negative views about these other groups, rather than obesity *per se*. In an experimental German study, subjects expressed higher fat phobia in response to a vignette of a low-SES person with obesity, compared to a high-SES person(56), suggesting negative views about multiple groups may jointly influence reactions. Cultural critics and social scientists have long argued that in popular discourse obesity and poverty are closely connected, and that this link is moreover heavily moralized. Hester & Walters(57) describe a conflation of poverty, body size, and immorality, in which “*the unwillingness or inability to regulate one’s body size is seen as a particularly classed form of failure, frequently linked with perceived laziness or ineptitude*”. This narrative is prominent in mass media: for instance, in 2015 one of the UK’s highest-circulation newspapers published the following two articles three days apart: *"We'd rather be fat on benefits than thin and working': Mother and daughter who weigh a total of 43 STONE and boast matching mobility scooters receive £34,000 a year in handouts’*(58) and *‘Now we're paying disability benefit to obese under 5s! Outrage after official figures show pre-school children are getting handouts’*(59). In recent years, an important development has been the rise in television documentaries which follow people on low incomes, often presenting them in negative terms(60) including by highlighting the size of participants’ bodies(61). Neither is this perspective restricted to the producers: analysis of tweets by viewers of one such programme, *Benefits Street*, highlighted that they frequently contained the words “fat” and “lazy”(62). Prominent politicians have contributed to this discourse: MPs have referenced the “bone idle” protagonists of *Benefits Street* to argue for reform of benefits(63), reflecting a wider, strategic use of stigma in political rhetoric(64). In a speech as leader of the opposition, ex-prime minister David Cameron explicitly linked obesity with poverty in a moral framework, grouping them with alcohol and drug addiction as social problems which “are often the consequence of the choices people make.”(65)

In this context, it is plausible that a person’s views about obesity may be related to their views about welfare recipients. To our knowledge, this has never been explored using survey data. However, a US study reported a connection between attitudes about obesity and about people affected by poverty(49). Moreover, evidence suggests that a person’s views about welfare recipients are in turn underpinned by their core political values: how right-wing (as opposed to left-wing) and how authoritarian (as opposed to libertarian) they are. Widely regarded as the two fundamental dimensions of political conflict, the former can be interpreted as concerned with equality and redistribution in the economic sphere, and the latter with personal freedom and tolerance for diversity in non-economic domains(66). In many countries, more highly educated people view welfare recipients less negatively than people with fewer qualifications(67,68) and this is explained more by education differences in libertarian-authoritarian values than in left-right values. This suggests that for many people welfare dependence is conceptualized not primarily as an economic problem, but rather as a transgression of social norms, a kind of moral deviance(69). With obesity squarely conceptualized and discussed as a moral issue(65,70), it is plausible that these values may be similarly linked to weight-stigmatizing attitudes. However, research into the influence of political values on weight-related attitudes is scarce, US-focused, and has typically treated political values as unidimensional(50,51,71).

In this context, we explore predictors of weight-stigmatizing attitudes in a nationally representative sample of British adults (N=2186). We consider demographic and socioeconomic characteristics, factors related to one’s own BMI and health, beliefs about the causes and consequences of obesity, and two dimensions of core political values. Finally, we examine associations with views about welfare recipients, a stigmatized group frequently associated with obesity.

## Methods

### Participants

The British Social Attitudes Survey (BSAS) is a nationally representative, annual survey of around 4000 British adults(72) capturing public attitudes on social and political issues. Each year, core modules are completed by all participants, and other modules completed by a random half of participants. In 2015, half the sample (N=2188) were asked to complete a module on obesity, including questions designed to capture stigmatizing attitudes. Two participants lacking data on age were excluded, leaving an analytic sample of N=2186.

### Measures

#### Outcomes

The main outcome was an index of weight-stigmatizing attitudes, based on participants’ agreement with the following statements: *1) Most very overweight people are lazy. 2) Most very overweight people could lose weight easily if they tried. 3) People who are very overweight should have the same right as anyone else to receive expensive NHS treatments. 4) People who are very overweight care just as much about their appearance as anyone else*. For each, participants could respond from 1 (agree strongly) to 5 (disagree strongly). A small number of participants answered *“can’t choose”* (between 1.1% for item 3 to 2.7% for item 4); these responses were recoded to missing. Adding these responses resulted in a single index (Cronbach’s alpha: 0.65) which was standardized for analysis. Additional analyses examined associations with each of the four items individually. As a secondary outcome, we considered responses to a question asking participants how they would feel if a close relative *‘married or formed a long-term relationship with somebody who is very overweight’*. Participants could answer with *“The person’s weight would affect how I felt about the relationship at least a little”, “The person’s weight would make no difference to how I felt”*, or *“can’t choose”* (10.3%). This item was coded for analysis as an ordered categorical variable, with *“can’t choose”* as the middle group.

#### Demographic and socioeconomic predictors

Gender was based on self-reported information. To allow for nonlinear effects, age was categorised in roughly 10-year bands: 18-29, 30-39, 40-49, 50-59, 60-69, 70-79, 80-97. Self-reported ethnicity was classified as white (88.9% of the sample), Black (3.1%), Asian (5.7%) and mixed or other (2.1%). Different socioeconomic indicators are known to have different associations with both social and political attitudes(73,74) and with health(75). We therefore explored associations with educational attainment, income, occupational social class, and employment status, treating all variables as categorical to allow for nonlinear effects. Ordered socioeconomic indicators were coded to have the least advantaged group as the reference category, except for education, where this group was substantially smaller than the others. Highest educational qualification was categorized as university degree, qualifications below degree, and no qualifications. Total household income was included in tertiles. We also considered subjective income, based on participants’ responses to the question *“Among which group would you place yourself: high income, middle income, or low income?”*. For occupational social class, we used the National Statistics Socio-Economic Classification (NS-SEC), in six groups: Higher managerial, professional, and administrative occupations; intermediate occupations; small employers and own account workers; lower supervisory and technical occupations; semi-routine and routine occupations, and a small group who could not be classified(76). Employment status was categorized as employed, unemployed, permanently out of work due to sickness or disability, and other.

#### Own BMI and health

BMI in kg/m^2^ was calculated based on self-reported weight and height and in main analyses treated as continuous. Additional analyses explored associations with BMI categories (<18.5, 18.5-24.9, 25.0-29.9, 30.0-34.9, 35+). Participants stated their perception of their own weight, choosing from: very underweight, underweight, about the right weight, overweight, or very overweight. Due to small cell sizes, the two underweight categories were merged. Participants also reported how happy they were with their shape, choosing from: very happy, happy, neither happy nor unhappy, unhappy, and very unhappy; this was treated as continuous in analysis. We also included a binary measure of whether participants reported a long-term illness or disability.

#### Beliefs about the causes of obesity

A series of questions explored participants’ beliefs about the causes of obesity, with participants asked to state their agreement from 1 (agree strongly) to 5 (disagree strongly) with a list of statements. Four questions explored how much a participant thought obesity was due to inheritance, low metabolism, diet, or exercise: “*Being overweight is something you inherit from your parents*”, “*Most overweight people have put on weight due to low metabolism*”, “*Most people who are overweight have put on weight due to eating too much*” and “*Most people who are overweight have put on weight due to lack of exercise*”. After reverse-coding, agreement with these statements was added up into a single index, capturing how much participants saw obesity as a matter of inheritance or metabolism rather than diet or exercise. Six statements gauged a respondent’s perception of structural or environmental factors which might restrict a person’s ability to maintain a healthy weight: *“Healthy food is too expensive for most people”, “Most people lack time to make healthy meals”, “Finding time to be physically active is difficult for many people”, “Everyday life nowadays means people end up spending too much time sitting down”, “Generally, there are not enough safe places to walk or cycle in”*, and *“Cheap fast food too easily available”*. Agreement with these statements was added up into a single index capturing perceived importance of these restrictions. Both indexes were standardized for analysis.

#### Beliefs about consequences of obesity

Participants were asked whether they thought overweight people were more likely to suffer from a list of common health conditions, from heart disease and arthritis to depression and asthma. Positive responses were added up into a scale ranging from 0 to 11, indexing perceived health consequences of obesity. This was standardized for analysis.

#### Social and political attitudes

Each year BSAS participants answer questions designed to capture left-right and libertarian-authoritarian values. Left-right values were measured by agreement from 1 (agree strongly) to 5 (disagree strongly) with the following five statements: “*Government should redistribute income from the better-off to those who are less well off*”, “*Big business benefits owners at the expense of workers”, “Ordinary working people do not get their fair share of the nation’s wealth”, “There is one law for the rich and one for the poor”*, and *“Management will always try to get the better of employees if it gets the chance.”* Libertarian-authoritarian values were measured by agreement from 1 (agree strongly) to 5 (disagree strongly) with the following six statements: *“Young people today don’t have enough respect for traditional British values*”, “*People who break the law should be given stiffer sentences”, “For some crimes, the death penalty is the most appropriate sentence”, “Schools should teach children to obey authority”, “The law should always be obeyed, even if a particular law is wrong”* and *“Censorship of films and magazines is necessary to uphold moral standards.”* We used the derived indexes, which are coded such that higher values correspond to more right-wing and more authoritarian views. Both were standardized for analysis. Building on previous work(69), agreement with the following statements was used to capture negative attitudes towards welfare recipients: *“Around here, most unemployed people could find a job if they really wanted one”, “Many people who get social security don’t really deserve any help”, “Most people on the dole are fiddling in one way or another”* and *“If welfare benefits weren’t so generous, people would learn to stand on their own two feet.”* Agreement from 1 (agree strongly) to 5 (disagree strongly) with each statement was added up into a single index (Cronbach’s alpha: 0.85) which was standardized for analysis.

### Statistical analysis

Missing values were imputed using multiple imputation by chained equations (m=50), with age bands and gender on the right-hand side. For weight-stigmatizing attitudes, beliefs about the causes of obesity, and welfare-stigmatizing attitudes, individual items were imputed separately, and indexes constructed post-imputation. Categorical variables were imputed with logistic, ordered logistic or multinomial logistic regression, and continuous variables including BMI with truncated regression or predictive mean matching. Separate imputation models were run to explore associations with categorized BMI, with BMI groups imputed using multinomial logistic regression. Multivariate regression was used to examine associations of weight-stigmatizing attitudes with demographic and socioeconomic factors, ethnicity, factors related to a person’s own BMI and health, beliefs about the causes of obesity, and social and political attitudes. Gender and age effects were mutually adjusted. Models exploring associations with all other predictors included gender and age bands as covariates, but not each other. Consequently, differences by education, income, occupational social class, and employment status do not adjust for other dimensions of socioeconomic position. To explore whether sociodemographic differences were explained by other factors, potential mediators were added to regression models as covariates and attenuation of sociodemographic differences observed. In additional analyses, ordered logistic regression was used to explore predictors of a secondary outcome: how participants would feel if a close relative married or formed a relationship with someone very overweight. Lastly, models were run to explore associations with each of the four items comprising the index of weight-stigmatizing attitudes as separate outcome variables. All code used in analysis is available at https://github.com/ammhughes/predictors_of_weight_stigmatizing_attitudes/.

## Results

Of 2186 participants in the analytic sample, 1226 (56.1%) had complete data on all variables used in analysis. T-tests and chi-squared tests showed that compared to participants who had missing data, they were more likely to be male, white, and in employment (all p<0.05). They were more highly educated (e.g., 26.6% vs 19.4% with a degree), had higher objective and subjective income, and tended to be in more advantaged NS-SEC groups. They had slightly higher BMI (26.7kg/m^2^ vs 26.0kg/m^2^), thought inheritance or metabolism was less important in causing obesity, placed less importance on structural factors relevant to obesity, and thought the health consequences of obesity were slightly greater (all p<0.05). They did not differ in terms of age or the weight-stigmatizing index. They were less likely (7.6% vs 18.7%) to respond “can’t choose” to the item asking if their relative’s partner’s weight would affect how they felt about their relationship. Characteristics of the sample prior to imputation are provided in supplementary table S1, and the proportion of each variable imputed in supplementary table S2.

### Outcome: index of weight-stigmatizing attitudes

**Figure 1:**
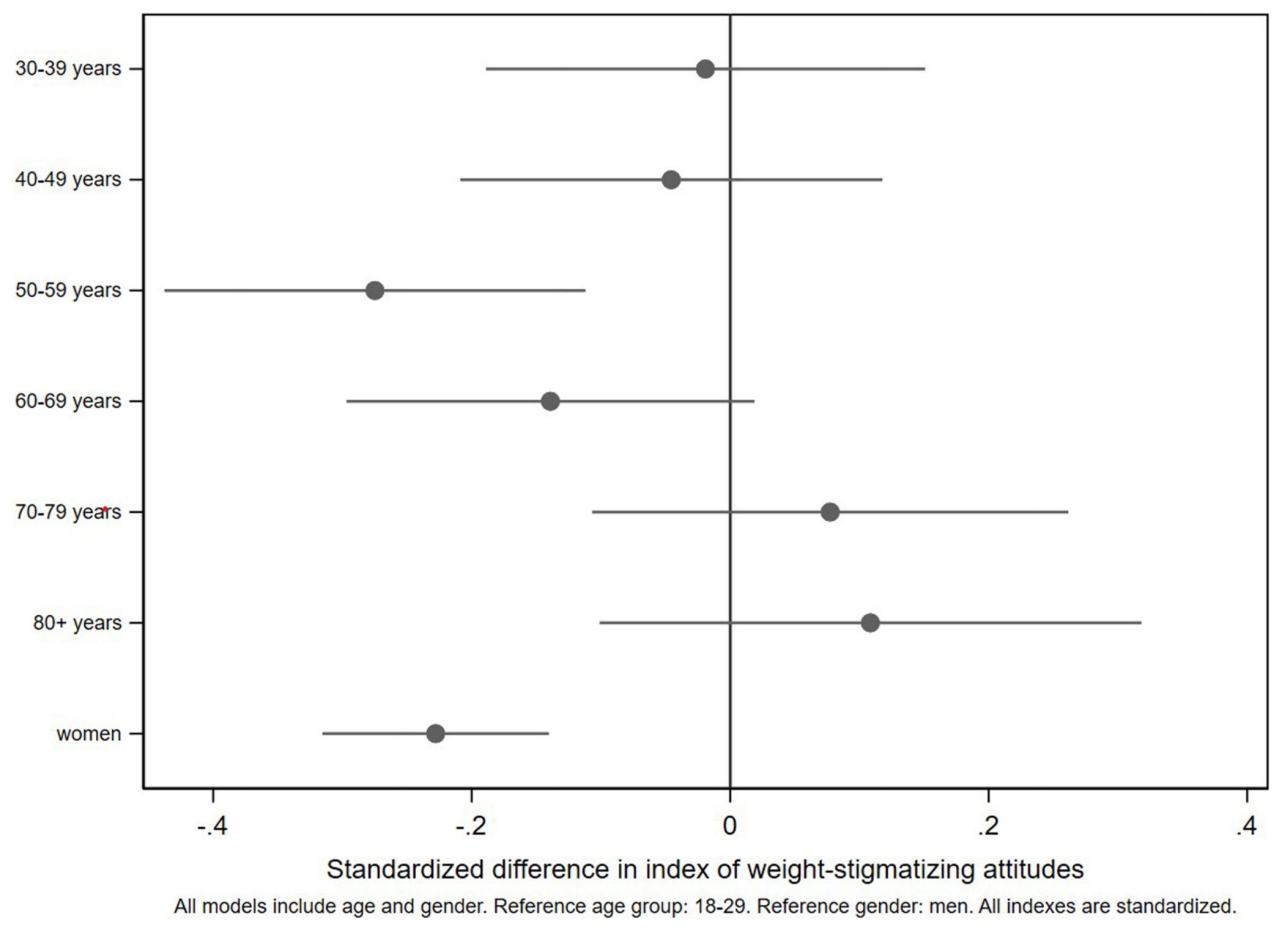
demographic differences in the weight-stigmatizing index

**Figure 4:**
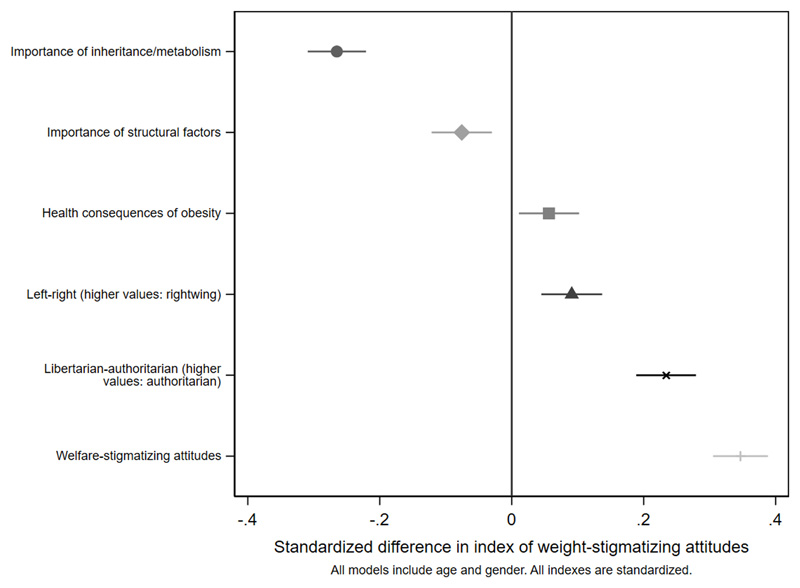
beliefs and attitudes: differences in the weight-stigmatizing index

Associations with the standardized index of weight-stigmatizing attitudes are shown in Figures 1 [Fig F2] [Fig F3]-4 and Table S3. Women were less weight-stigmatizing than men (beta: -0.23 S.D., CI: -0.32, -0.14). Age showed a nonlinear relationship, with the least weight-stigmatizing attitudes in the middle age groups. Consistent with previous work using this survey(39), there was little evidence of differences between ethnic groups (Table S3). Compared to people with a university degree, people with qualifications below a degree had more weight-stigmatizing attitudes (beta: 0.18, CI: 0.07,0.29), but people with no qualifications did not (beta: 0.04, CI: -0.10,0.13). Compared to the lowest tertile of objective household income, weight-stigmatizing attitudes were raised in both the middle (beta: 0.12, CI:0.00,0.25) and highest tertiles (beta: 0.10, CI: -0.03,0.22). Point estimates suggested subjective income was positively associated with weight-stigmatizing attitudes, and that intermediate NS-SEC groups had the most weight-stigmatizing attitudes, but confidence intervals for most coefficients were consistent with no differences. There was little evidence of any differences by employment status.

As expected, a person’s own BMI was negatively associated with their weight-stigmatizing attitudes (beta: -0.22, CI: -0.26,-0.18 per 5kg/m^2^). Additional analyses in which BMI was categorized (Table S3) indicate a linear relationship. Reporting a long-term illness or disability was negatively associated with weight-stigmatizing attitudes (beta: -0.26, CI:-0.36,-0.17). Happiness with one’s own weight was associated with weight-stigmatizing attitudes (beta: 0.13, CI: 0.08,0.17). People who viewed their weight as about right scored highest for weight-stigmatizing views: this was lower for people who saw themselves as underweight (beta:-0.16, CI:-0.34,0.02) a bit overweight (beta:-0.25, CI:-0.34,-0.15), or very overweight (beta: -0.83, CI:1.00,-0.65). People who felt that obesity was mostly due to inheritance or metabolism rather than diet or exercise had less weight-stigmatizing views (beta: -0.27, CI: -0.31,-0.22), as did people who felt that structural factors restrict people’s choices around diet and exercise (beta: -0.08, CI: -0.12,-0.03). People who thought obesity had more negative health consequences had slightly more stigmatizing views (beta:0.06, CI:0.01,0.10). Participants who were more right-wing had more weight-stigmatizing attitudes (beta: 0.09, CI: 0.04,0.14), as did participants with more authoritarian as opposed to libertarian values, where the association was stronger (beta: 0.23, CI: 0.19,0.28). Of all predictors, the strongest association was seen with the welfare-stigmatizing index (beta: 0.35, CI: 0.31,0.39).

**Figure 6:**
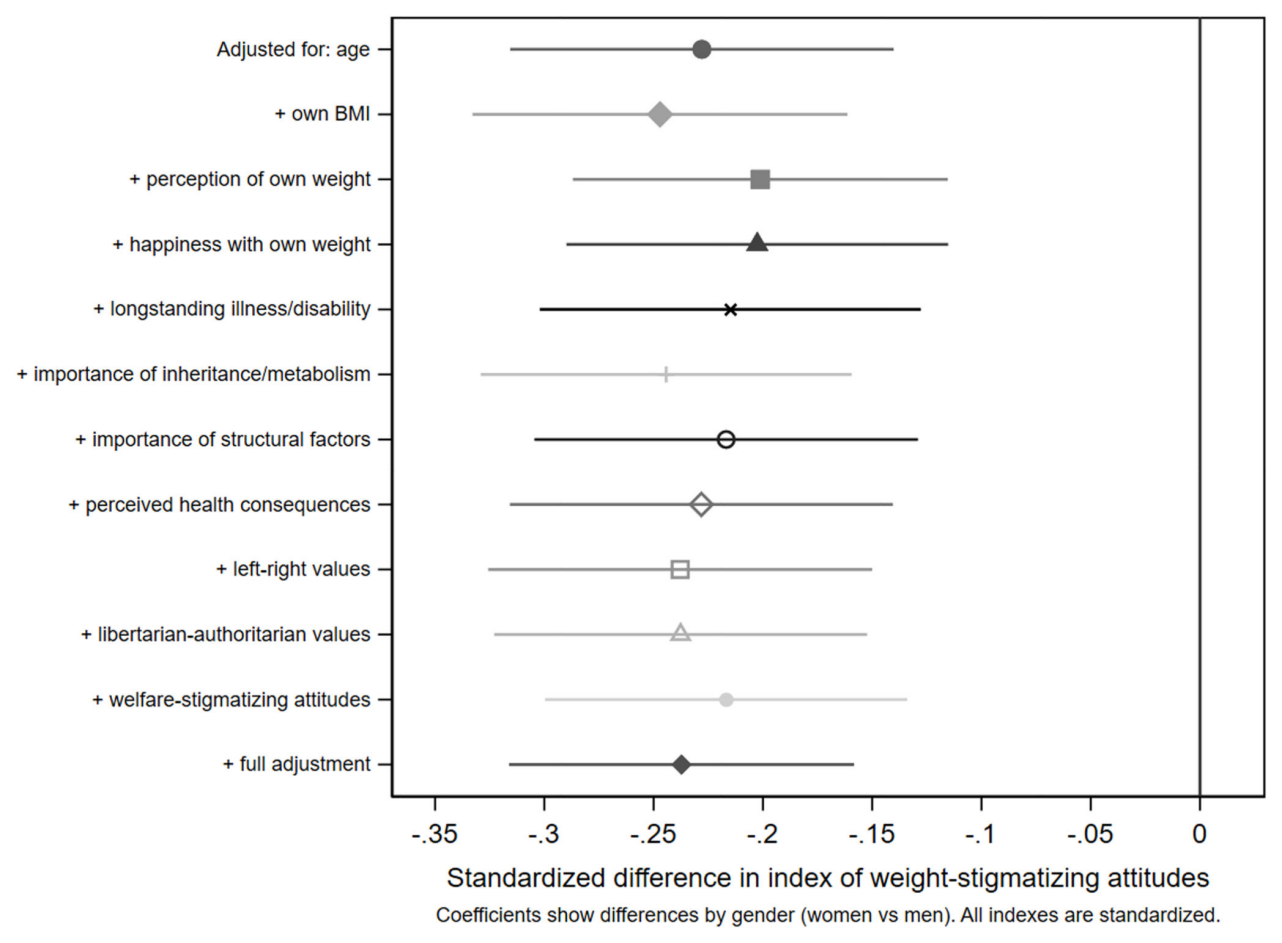
attenuation of gender differences in the weight-stigmatizing index

**Figure 7:**
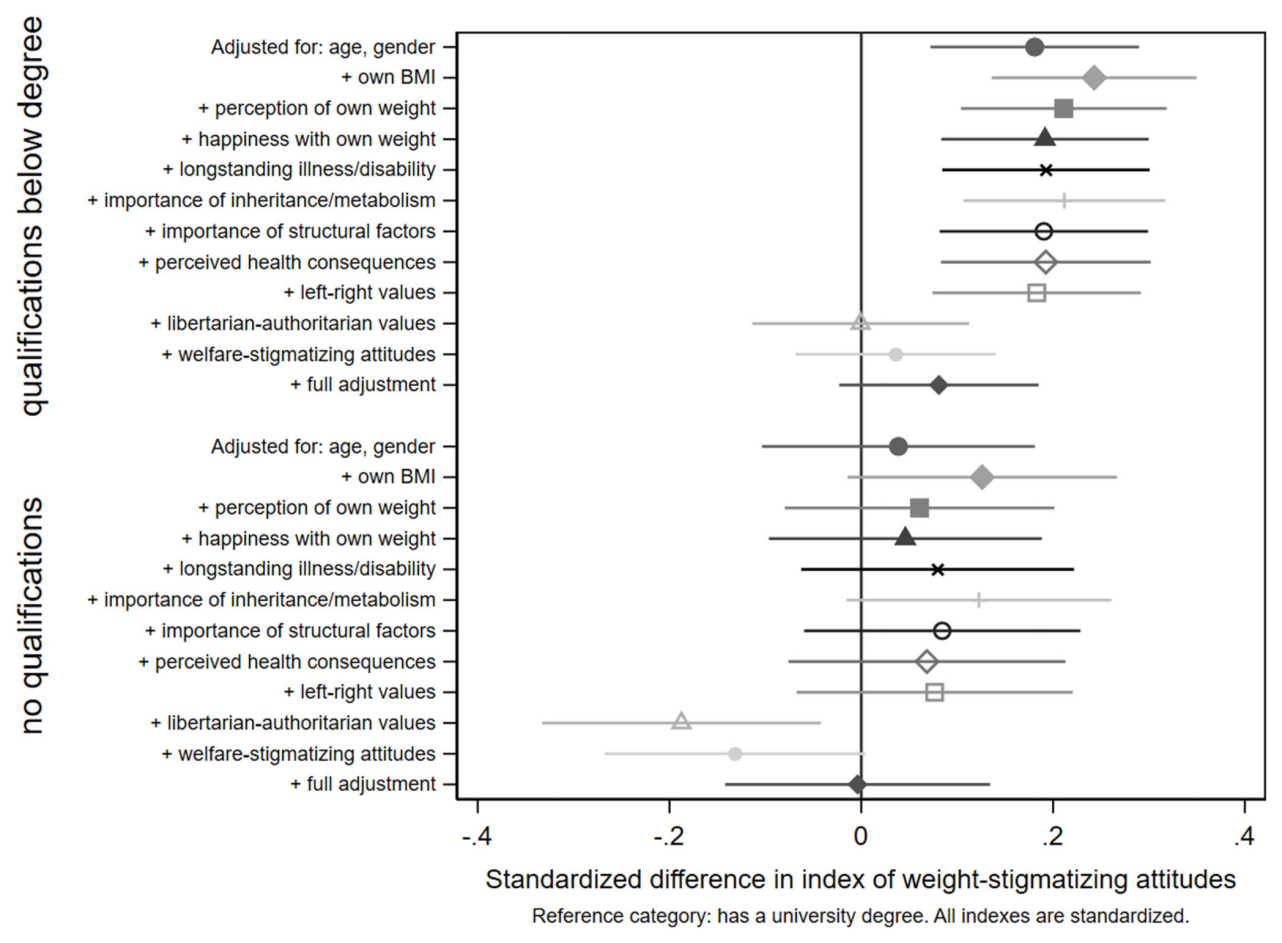
attenuation of education differences in the weight-stigmatizing index

**Figure 8:**
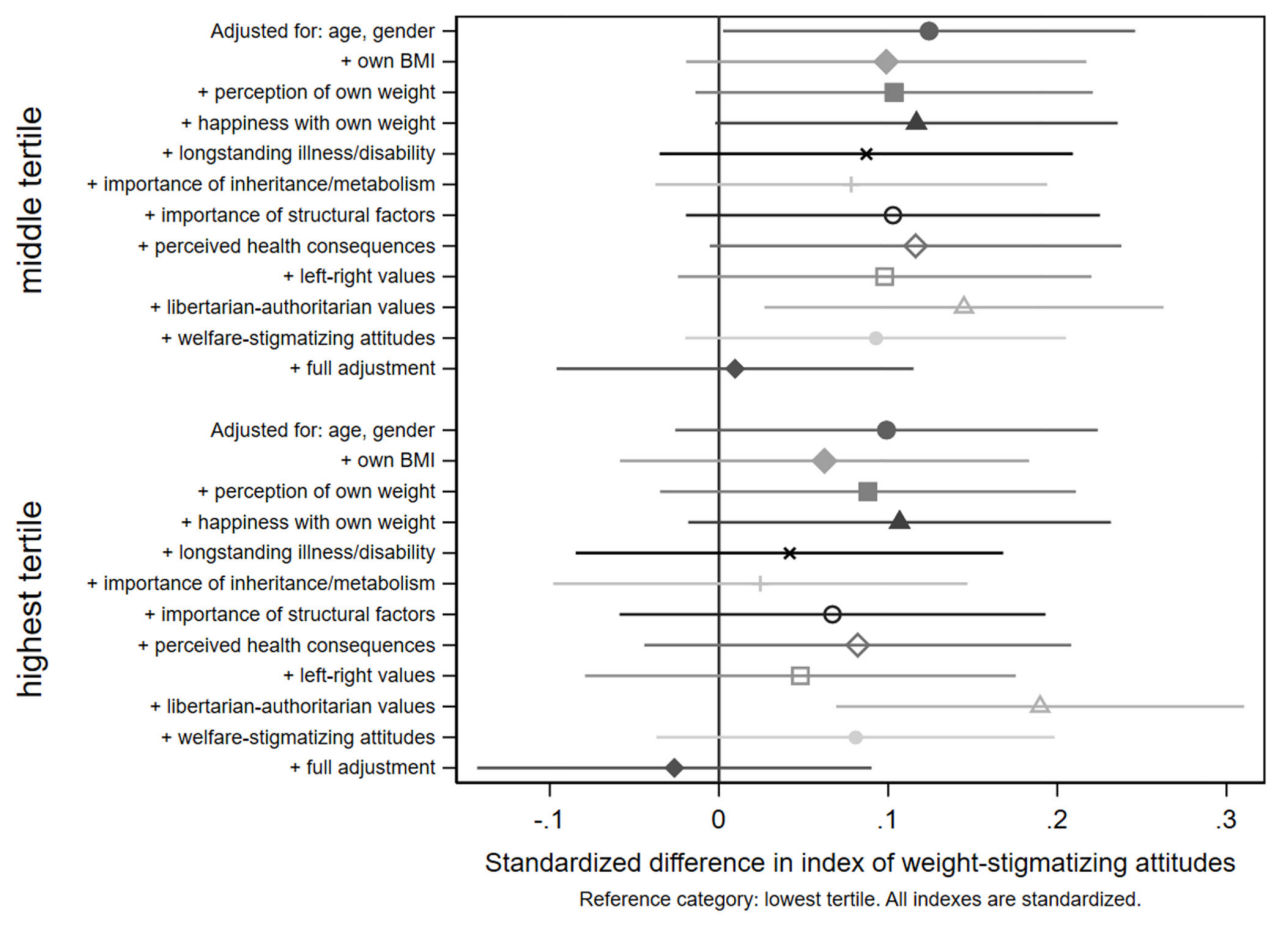
attenuation of differences by household income tertile in the weight-stigmatizing index

**Figure 9:**
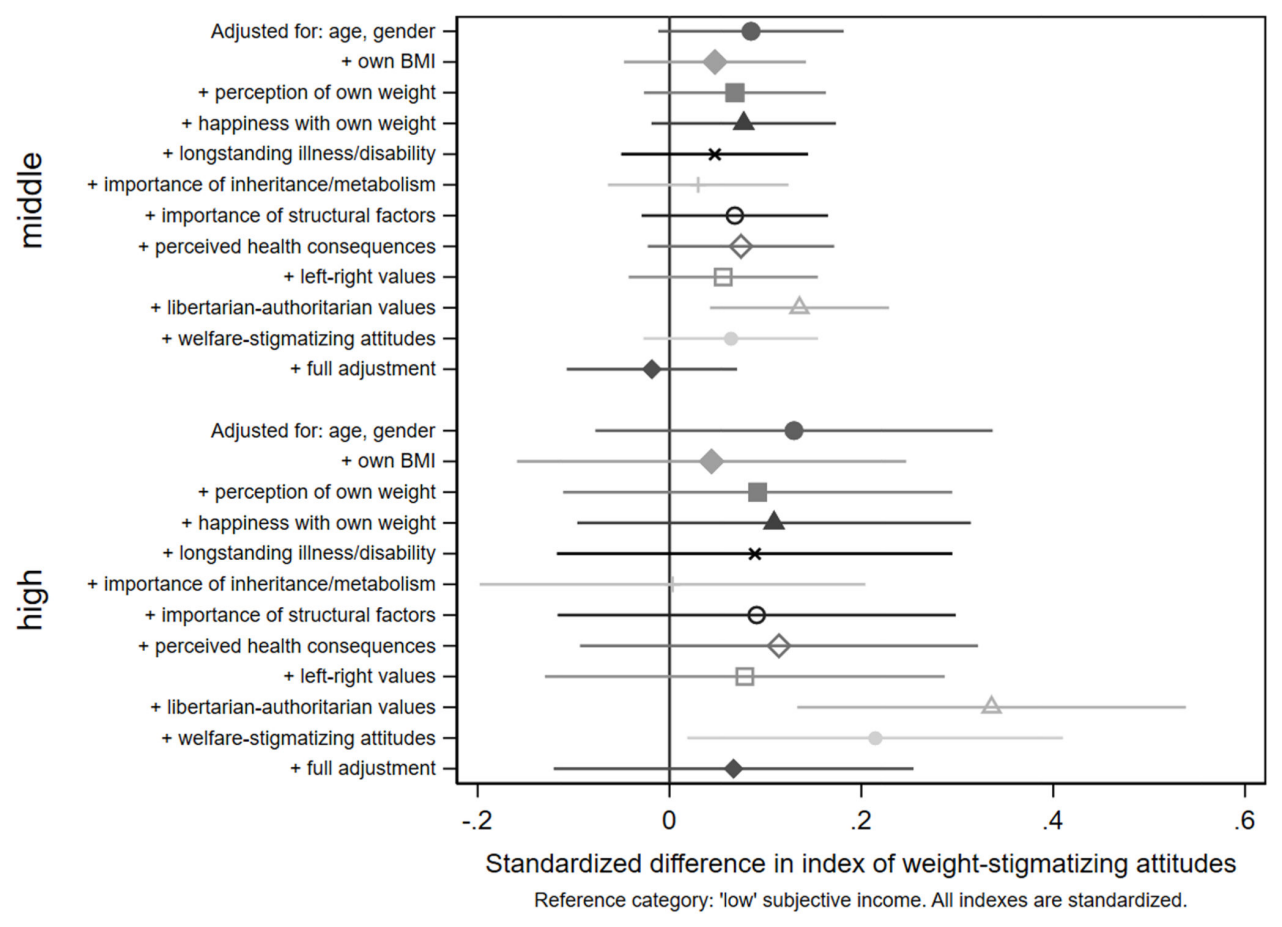
attenuation of differences by subjective income in the weight-stigmatizing index

Age differences in weight-stigmatizing attitudes were attenuated with adjustment for own BMI and views about own weight (Figures 5a and 5b, Tables S4a and S4b). In contrast, adjustment for health-related factors, beliefs, and values individually or together had limited impact on gender differences (Figure 6, Table S5), suggesting that they did not substantially explain them. For educational qualifications (Figure 7, Table S6), adjustment for own BMI and perception of own weight seemed to increase group differences (e.g., beta for the middle group: 0.24, CI: 0.14,0.35). Individual adjustment for libertarian-authoritarian values, and for welfare-stigmatizing views, fully explained the more weight-stigmatizing attitudes in the middle group compared to participants with a degree, whilst also revealing less weight-stigmatizing attitudes among people with no qualifications, once either factor was controlled for (e.g., -0.19, CI:-0.33,-0.04 adjusted for libertarian-authoritarian values). Including all factors together, educational differences were fully attenuated. For objective and subjective income (Figures 8-9, Tables S7-S8), most factors led to modest attenuation of differences. An exception was libertarian-authoritarian views, where adjustment increased the difference for the middle groups, and revealed more weight-stigmatizing attitudes in the most advantaged groups once libertarian-authoritarian views were accounted for (for objective income: beta;0.19, CI:0.07,0.31, for subjective income: beta:0.34,CI:0.13,0.54). Including all factors together, differences were fully explained.

### Outcome: relative’s partner’s weight would affect how I feel about the relationship

Associations with this outcome are shown in Table S9. Women were less likely than men to say that their relative’s partner’s weight would affect how they felt about the relationship (OR:0.80, CI:0.67,0.97) and older people were generally more likely to say so than younger people (e.g., OR: 1.77, CI:1.23,2.56 for the 70-79 age group). For education, objective income and subjective income, there were clearly increasing odds in more advantaged groups (e.g., compared to participants with a degree, OR:0.63, CI:0.50,0.78 and OR:0.39, CI:0.29,0.53 for the middle and lowest education groups). For occupational social class, odds were generally lower for less advantaged groups. There was no strong evidence of differences between ethnic groups or by employment status.

People whose own BMI was higher were less likely to say that their relative’s partner’s weight would affect how they felt about the relationship (OR:0.66, CI:0.59,0.73 per 5kg/m^2^). This was also the case for people who considered themselves overweight or very overweight, and people who placed greater importance on inheritance/metabolism and structural factors. Happiness with one’s weight was positively associated with the outcome, as was belief that obesity has more negative health consequences. Having a long-term illness or disability, left-right views and welfare-stigmatizing views did not appear associated with the outcome. People with more authoritarian views were less likely to say that their relative’s partner’s weight would affect how they felt about the relationship (OR:0.91, CI:0.83, 1.00).

### Associations with individual index items

We considered as individual outcomes the index’s four items: *“Most very overweight people are lazy”, “Most very overweight people could lose weight easily if they tried”, “People who are very overweight should have same rights as anyone else to receive expensive NHS treatments*” (reverse-coded) and *“People who are very overweight care just as much about their appearance as anyone else*” (reverse-coded), hereafter *lazy*, l*ose*, *nhs*, and *care*. These showed divergent associations with several predictors, with *nhs* acting differently to the other three (Supplementary Figure S1). Notably, there were clear gender differences in *lazy, lose* and *care*, with women scoring lower for all three items, but no gender difference in *nhs*. For objective income, subjective income, and the middle education category, there was a greater difference in *nhs* than the other items. Three groups scored higher for lazy and lose but lower for *nhs* and *care:* participants with no qualifications, NS-SEC groups 6&7, and Asian participants. Perceived importance of structural factors was negatively associated with *nhs*, and perceived health consequences positively associated with *nhs*. The left-right index was most associated with *nhs*, while the libertarian-authoritarian index and the welfare-stigmatizing index were more clearly associated with *lazy* and *lose*.

## Discussion

In a representative sample of British adults, we found that women, people in their 50s, people with a higher BMI, and people who were less satisfied with their own weight were less weight-stigmatizing. Weight-stigmatizing-attitudes were negatively associated with the belief that obesity is influenced by factors outside an individual’s control, and positively associated with a person’s perception of the negative health consequences of obesity. An index of weight-stigmatizing attitudes was positively associated with income, while for education and occupational social class, it was highest in intermediate groups. The index was positively associated with right-wing values, but more strongly associated with authoritarian values and a measure of welfare-stigmatizing attitudes. Libertarian-authoritarian values influenced socioeconomic differences, such that conditional on these values, participants with no qualifications were less stigmatizing than those with a degree. However, this was balanced by socioeconomic differences in perceived causes of obesity and other factors, and with full adjustment no education and income differences remained. Age differences were somewhat explained by own BMI and views of own weight. In contrast, lower weight-stigmatizing attitudes among women were not explained by any of the factors considered, suggesting this pattern has other causes. Research indicates that women are more likely to experience weight stigma at a given weight(28,77,78), and increased awareness of these processes may account for the difference.

Our findings for gender are consistent with most studies on explicit weight bias(34,36,37), but contrast with a representative German study in which no gender difference was detected(35). We found the least weight-stigmatizing attitudes among people in their 50s; other studies have reported positive(35), negative(37,39) or no clear association(36) with age, but nonlinear effects were not always considered(33,36). Our findings regarding beliefs about the causes of obesity accord with extensive evidence that stigmatization of obesity(35,36,42) and stigmatization more widely(3), are strongly linked to perceptions of responsibility. Results are also broadly in line with evidence on implicit weight bias, where men also have been found to score higher(13,51,79) but where, compared to explicit attitudes, smaller or null results for gender and other differences have been reported(37,51). It has been argued that results for explicit and implicit measures diverge because the latter is less affected by social desirability bias(37). However, it remains unclear to what extent implicit measures are biased by other processes, and how much they succeed at capturing constructs relevant to behaviour(80). For both reasons, we position our findings principally with respect to the literature on explicit weight-related attitudes, to which our results contribute.

The strong association observed in this study between the weight-stigmatizing and welfare-stigmatizing indexes suggests that these attitudes do not arise independently. Like previous research on welfare stigma, we found that while people with higher incomes are more weight-stigmatizing, highly educated people are less weight-stigmatizing. Moreover, both sets of attitudes were linked less to left-right values than to libertarian-authoritarian values(69). Our results therefore suggest not only that weight stigma and welfare stigma are connected, but that they may share common roots in fundamental political values. This may centre on the perceived moral importance of work or effort, which would accord with reported links between views about obesity and the Protestant work ethic (48,49), and evidence that people who lose weight are judged differently if it was via surgery or diet and exercise(50). Our results may also reflect the consequences of a political framing of welfare dependency, and obesity, as similar kinds of moral failure(65). Work comparing societies in which recent political discourse has differed, or qualitative research exploring how people understand their views in relation to other beliefs, could further unravel these findings.

Participants’ agreement with the statement *“People who are very overweight should have the same rights as anyone else to receive expensive NHS treatments*” behaved differently to the other three items in the index. This was the item most clearly associated with left-right values, which perhaps is unsurprising as it relates to distribution of resources. Conversely, the statements *“Most very overweight people are lazy”* and *“Most very overweight people could lose weight easily if they tried”* were the items most clearly associated with the libertarian-authoritarian index and the welfare-stigmatizing index. As with the full index, these associations suggest that perceived moral deviance in non-economic domains, or ethical importance placed on work or effort, may link authoritarian values, weight-stigmatizing attitudes, and welfare-stigmatizing attitudes. Divergent associations of *nhs* with other items is also consistent with the possibility that disagreement with a system which encourages people to claim welfare is distinct from dislike of the individuals who do so (81).

As a secondary measure of weight-stigmatizing attitudes, we considered whether the weight of a participant’s relative’s partner would affect how the participant felt about the relationship. Compared to the index, this measure showed similar associations with gender, factors related to own BMI, and beliefs about causes and consequences of obesity. At the same time, it showed contrasting associations with political predictors and education, suggesting it captures overlapping but distinct constructs. Besides weight-stigmatizing attitudes, responses could depend on the nature of a person’s relationships with their relatives, or the typical body weight in a community. Follow-up work could explore this divergence using qualitative methods or investigate if associations differ between areas where prevalence of obesity differs.

Our findings have clear implications for initiatives aiming to tackle weight stigma. Results demonstrate that, at least for British adults, weight-stigmatizing attitudes are closely linked to a person’s demographics, political values, and wider social attitudes. Individual, interpersonal, and structural approaches(82) to weight stigma reduction may therefore be more effective if they acknowledge the political context in which weight stigma arises. Interventions to reduce weight stigma have often focused on “removing blame”(83) by changing beliefs about obesity’s causes, emphasising factors beyond an individual’s control(44–47). However, another approach is that of “drawing equivalences”. This involves underscoring commonalities between members of a stigmatized group and others, and demonstrating equivalence with respectable citizens, for instance by mobilizing individuals in the group with high social and cultural capital(83). This aligns with calls for public health messaging in which people with obesity are represented positively and in multiple roles, including in positions of power(84). Our results, which point to close links between attitudes about people with obesity and attitudes about other stigmatized groups, suggest weight stigma interventions which take this approach may be an effective complement to other anti-weight stigma strategies.

Findings also suggest that public support for policies relating to obesity may vary substantially with political attitudes on both the authoritarian-libertarian axis and the left-right axis. This includes laws prohibiting weight-related discrimination, which have more support among people who describe themselves as “liberal”(85), and healthcare policies which restrict access to services based on patients’ BMI. As of June 2021, 68% of local NHS commissioning bodies in England had implemented such policies for knee or hip arthroplasty(86), despite evidence that patients with a high BMI do not have poorer outcomes following knee arthroplasty(87). Similarly, those who see obesity as a moral failing may oppose policies aiming to mitigate obesity-associated harms, given that people are less supportive of harm reduction policies around behaviours which they morally object to (88,89). An example would be measures to reduce healthcare avoidance among people with obesity by making healthcare settings more welcoming(14). Advocates for specific public health interventions relating to obesity should be aware that support among the general public, political decision makers, and healthcare professionals may vary dramatically between people holding different sets of political attitudes that are linked to their perceptions of obesity.

### Strengths and limitations

A major strength of our study is use of a large, nationally representative sample, which has seldom been applied to study weight-stigmatizing attitudes. This is crucial: recent research on collider bias has shown that in nonrepresentative study samples, relationships between pairs of factors which jointly determine selection into the sample can be severely distorted(90). The richness of the data allowed investigation of a wide range of factors which were associated with weight-stigmatizing attitudes and contributed to their socioeconomic distribution. We considered multiple dimensions of socioeconomic position, finding that they related differently to weight-stigmatizing attitudes. Nevertheless, for ethnicity and occupational social class, small cell sizes limited the conclusions which could be drawn. Participants’ own BMI was calculated from self-reported height and weight, and associations with BMI based on measured height and weight may have differed. The study was cross-sectional, and multiple causal processes could give rise to the associations observed. Analysis with longitudinal data, examining changes over time in socioeconomic circumstances, BMI, health, and social attitudes could further unravel these processes. Factors not measured in this survey could confound associations: for example, personality traits could influence attitudes about obesity and political values(91). However, it is not clear that personality affects political values, rather than vice versa(92). Although we used the most recent data available with necessary measures, it was collected in 2015, and attitudes may have shifted since. However, the COVID-19 pandemic is unlikely to have reduced weight stigma(25) and despite many people claiming government assistance for the first time, its impact on attitudes to welfare appears limited(93). Moreover, cross-cohort and cross-national work suggests increasing prevalence of obesity does not necessarily reduce weight-stigmatizing attitudes(30,94).

Follow-up work could use methods including vignettes and qualitative approaches to better understand drivers of obesity-related attitudes, and why the index and the secondary outcome showed different socioeconomic and political patterning. These questions could be also examined using other measures of explicit weight bias. Another extension would be to look at associations with attitudes about mental illness (not possible in this survey, where no participants were administered both the mental health and obesity modules). A crucial question is whether these relationships are culturally specific to the UK. Cross-cultural work suggests that having a high BMI raises risk of depression in some cultures but protects against depression in others, depending on its cultural significance(95). Similarly, attitudes around obesity may differ in contexts where thinness, rather than obesity, have been historically linked to socioeconomic disadvantage and ill-health.

### Conclusion

Weight-stigmatizing attitudes are closely linked with political values and attitudes towards other stigmatized groups. Acknowledging these links may make efforts to tackle weight stigma, and other kinds of stigma, more effective.

## Supplementary Material

Supplementary material

## Figures and Tables

**Figure 2 F2:**
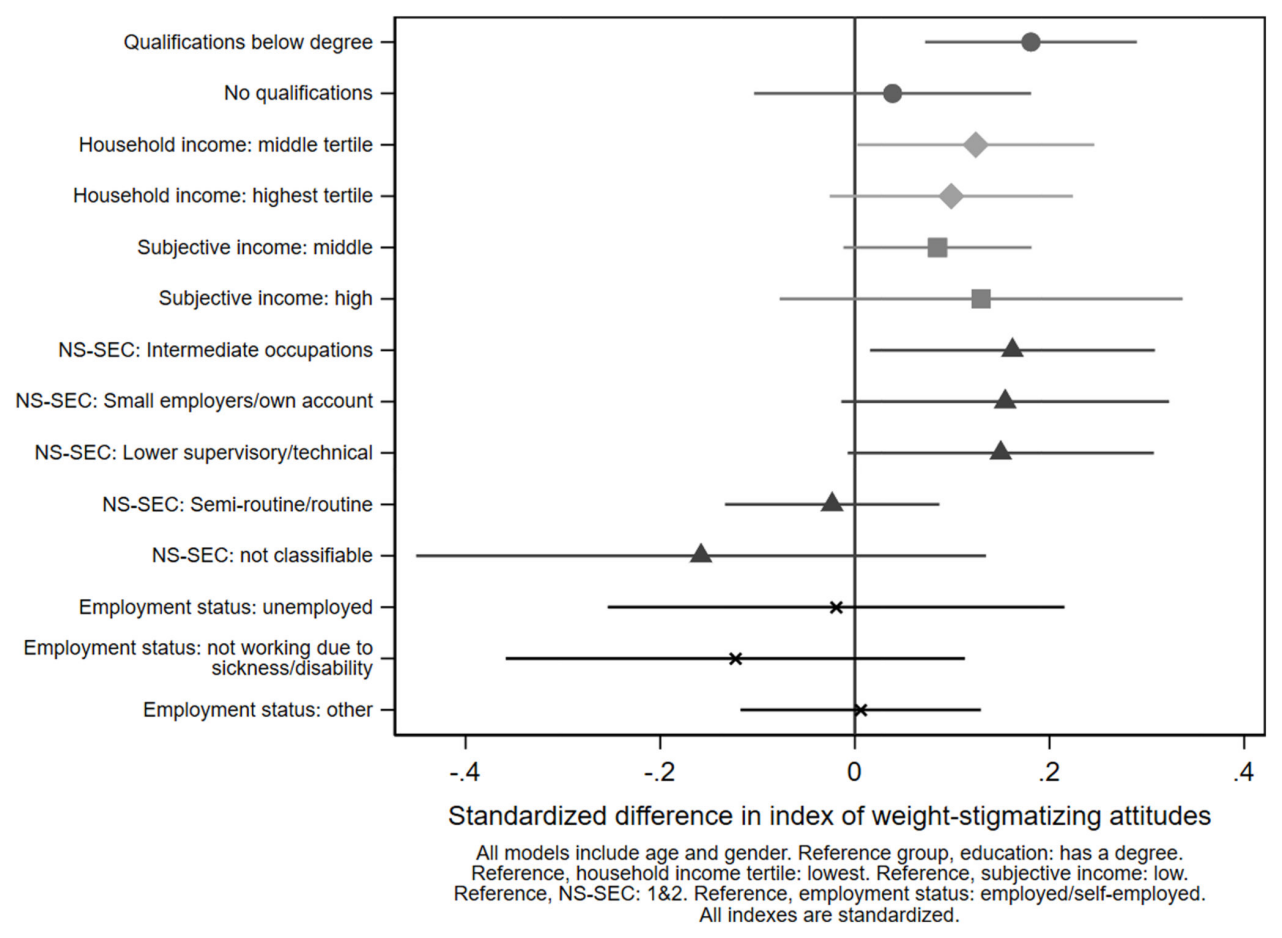
socioeconomic differences in the weight-stigmatizing index

**Figure 3 F3:**
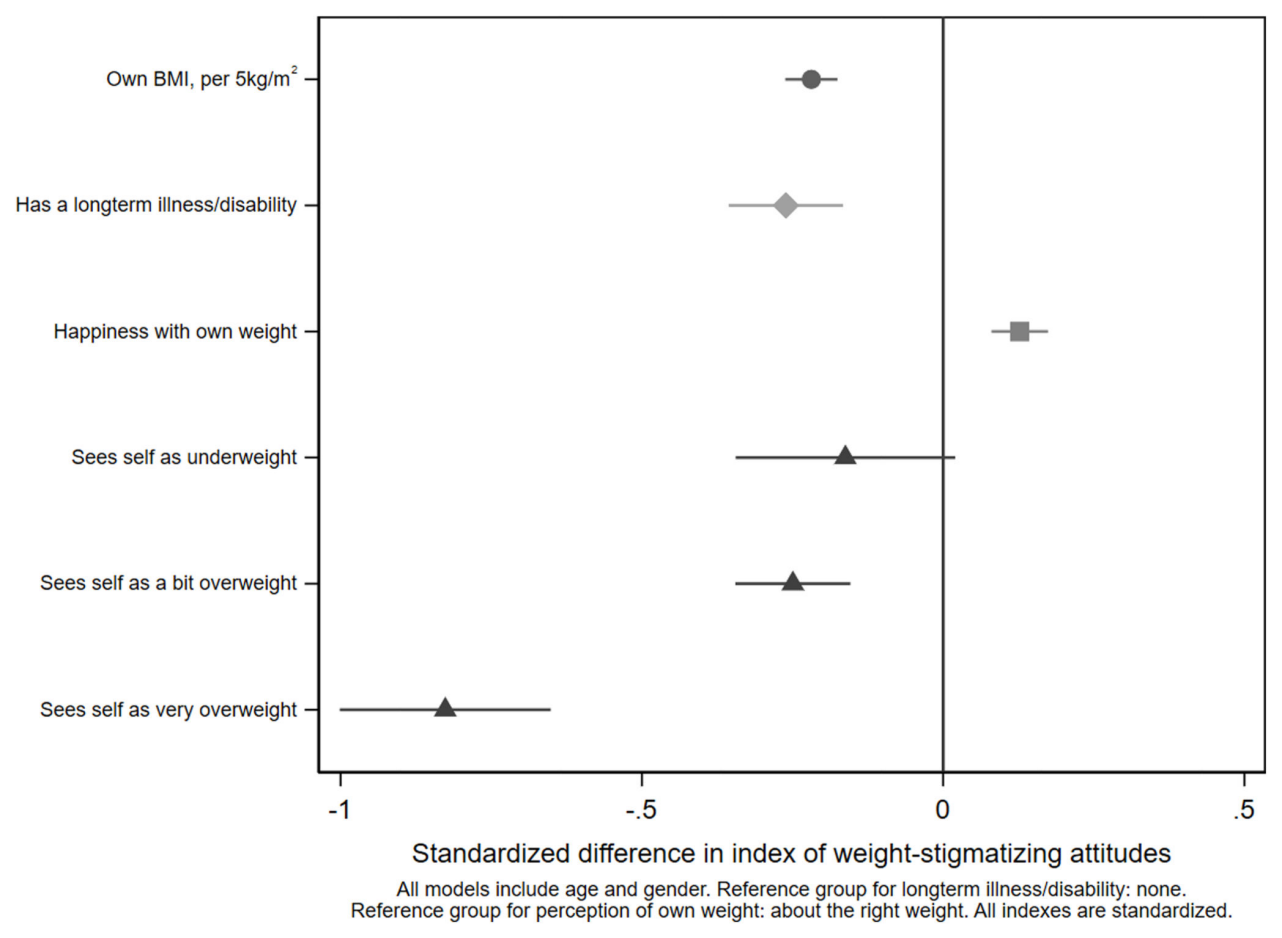
own weight and health: differences in the weight-stigmatizing index

**Figure 5a:**
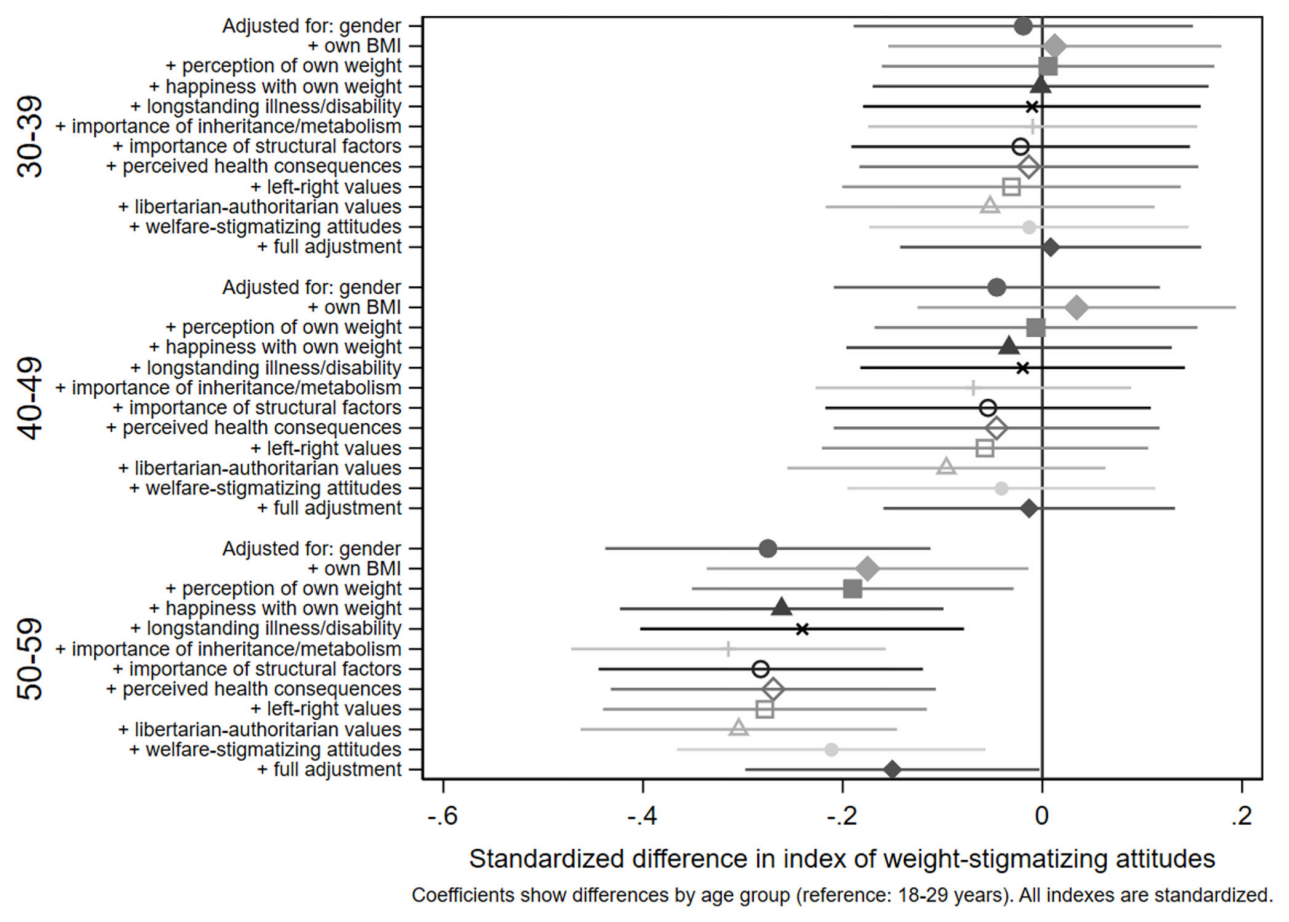
attenuation of age differences in the weight-stigmatizing index

**Figure 5b:**
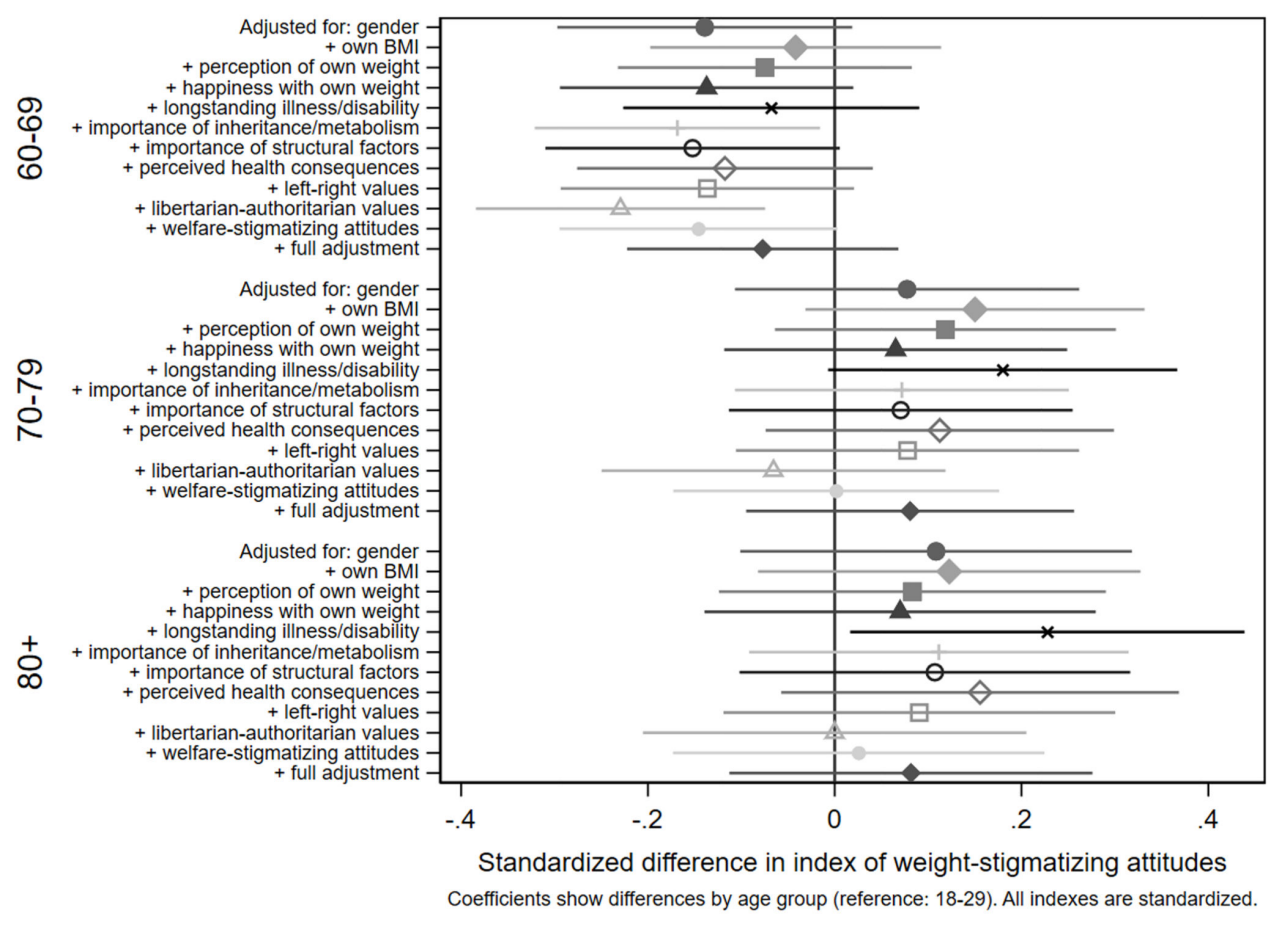
attenuation of age differences in the weight-stigmatizing index
