# Supplementary material for: Weight stigma, welfare stigma, and political values: evidence from a representative British survey
Source: Soc Sci Med. Author manuscript; Available in PMC 2024 Jan 6. (PMC7615479; doi:10.1016/j.socscimed.2023.116172)
Supplement: Supplementary material [file EMS192881-supplement-Supplementary_material.pdf]

Supplementary material: Weight stigma, welfare stigma, and political values: evidence from a representative British survey

Supplementary Figures:

Figure S1: Associations of all predictors with the weight-stigmatizing index and its individual items

Supplementary Tables:

Table S1: Descriptive characteristics of the sample, unimputed data

Table S2: Percent of variables imputed

Table S3: Associations from linear regression: index of weight-stigmatizing attitudes

Table S4a, S4b: Attenuation of age differences in the weight-stigmatizing index

Table S5: Attenuation of gender differences in the weight-stigmatizing index

Table S6: Attenuation of education differences in the weight-stigmatizing index

Table S7: Attenuation of objective household income differences in the weight-stigmatizing index

Table S8: Attenuation of subjective income differences in the weight-stigmatizing index

Table S9: Associations from ordered logistic regression: relative's partner's weight would affect how I felt about the relationship

Table S10. Associations from linear regression: individual items in the index of weight-stigmatizing attitudes

Figure S1: Associations* of all predictors with the weight-stigmatizing index and its individual items

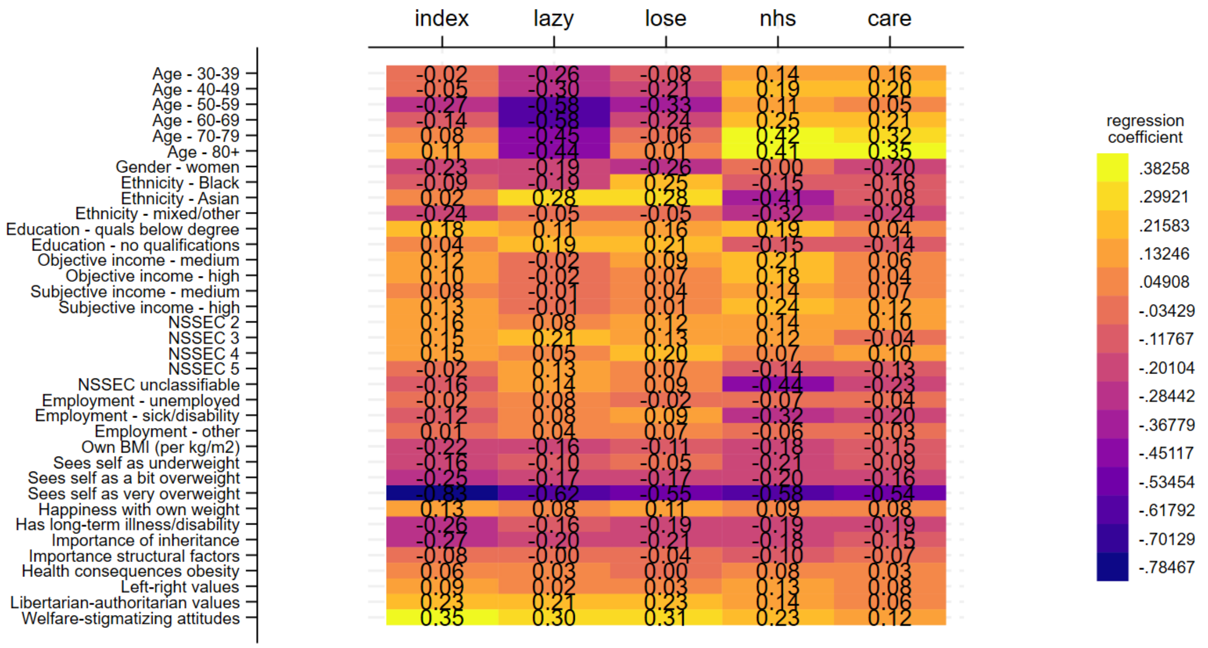

*All indexes, and all individual weight stigma items, are standardized.
All models include age and gender. Reference groups are age 18-29, men, white ethnicity, university degree, low objective income, low subjective income, NSSEC 1, employed, sees own weight as about right, no long-term illness/disability.

Table S1. Descriptive Characteristics of Analytic Sample, unimputed data (N=2186)				
**Continuous variables**		**mean**	**SD**	**N**
Age (years)		51.7	18.2	2,186
Body mass index (BMI), kg/m^2		26.4	5.2	2,012
Weight-stigmatizing views (possible range 0-16)		7.3	2.8	1,713
Causes of obesity: inheritance/metabolism (possible range 0-16)		5.3	2.4	2,140
Causes of obesity: structural factors (possible range 0-24)		14.0	3.3	2,168
Health consequences of obesity (possible range 0-11)		5.6	2.1	2,177
Left-right views (possible range 1-5)		2.6	0.8	1,824
Libertarian-authoritarian views (possible range 1-5)		3.6	0.7	1,832
Welfare-stigmatizing index (possible range 0-16)		8.7	3.3	1,798
**Categorical variables**	**Category**		**%**	
Gender	Men		45.4	2186
	Women		54.6	
Ethnicity	White		89.0	
	Black		3.1	
	Asian		5.7	
	Mixed or other		2.2	
Highest educational qualification	University degree or higher		23.4	2,183
	Qualifications below degree		55.2	
	No qualifications		21.4	
Household income tertile	Lowest		33.3	1800
	Middle		34.7	
	Highest		32.0	
Subjective income	Low		44.1	2,178
	Middle		50.8	
	High		5.1	
Occupational social class: NS-SEC	Higher managerial, professional, administrative		38.1	2,177
	Intermediate		12.0	
	Small employers/own account		8.5	
	Lower supervisory and technical		9.3	
	Semi-routine and routine		28.5	
	Unclassifiable		3.6	
Employment status	Employed		52.8	2,185
	Unemployed		4.4	
	Permanently sick/disabled		4.1	
	Other		38.8	
Long-term illness or disability	No		64.9	2,184
	Yes		35.1	
Perception of own weight	About the right weight		36.1	1,821
	Underweight or very underweight		6.6	
	A bit overweight		49.6	
	Very overweight		7.7	
Happiness with own weight	Very unhappy		3.9	1,829
	Unhappy		22.3	
	Neither unhappy nor happy		34.5	
	Happy		32.5	

	Very happy	6.7	
Close relative in a relationship with someone very overweight	Would not affect how I feel	62.6	1,812
	Would affect how I feel	26.8	
	Can't choose	10.5	

Table S2: Percent of values imputed*

	% imputed
Age	0
Gender	0
Own BMI (kg/m^2)	8.0
Weight-stigmatizing index	21.6
Happiness with own weight	16.3
Causes of obesity: inheritance/metabolism	2.1
Causes of obesity: structural factors	0.8
Health consequences of obesity	0.4
Left-right index	16.6
Libertarian-authoritarian index	16.2
Welfare-stigmatizing index	17.7
Ethnicity	0.1
Highest educational qualification	0.1
Household income tertile	17.7
Subjective income	0.4
Occupational social class: NS-SEC	0.4
Employment status	0.0
Perception of own weight	16.7
Long-term illness or disability	0.1
Relative's partner's weight would affect how I felt	17.1

*missing values were imputed using multiple imputation by chained equations (m=50).

Table S3. Associations from linear regression: index of weight-stigmatizing attitudes*

**Continuous variables**		**Coeff**	**CI**	**p**
Own BMI (per 5kg/m^2)		-0.22	-0.26,-0.18	<0.001
Happiness with own weight (per unit, range 1-5)		0.13	0.08,0.17	<0.001
Causes of obesity: choice vs biology (standardized)		-0.27	-0.31,-0.22	<0.001
Causes of obesity: structural factors (standardized)		-0.08	-0.12,-0.03	0.001
Health consequences of obesity (standardized)		0.06	0.01,0.10	0.02
Left-right values (standardized)		0.09	0.04,0.14	<0.001
Libertarian-authoritarian values (standardized)		0.23	0.19,0.28	<0.001
Welfare-stigmatizing attitudes (standardized)		0.35	0.31,0.39	<0.001
**Categorical variables**		**Coeff**	**CI**	**p**
Age group	18-29	Ref		
	30-39	-0.02	-0.19,0.15	0.83
	49-49	-0.05	-0.21,0.12	0.58
	50-59	-0.27	-0.44,-0.11	0.001
	60-69	-0.14	-0.3,0.02	0.08
	70-79	0.08	-0.11,0.26	0.41
	80+	0.11	-0.1,0.32	0.31
Gender	Men			
	Women	-0.23	-0.32,-0.14	<0.001
Ethnicity	White			
	Black	-0.09	-0.38,0.2	0.55
	Asian	0.02	-0.18,0.23	0.82
	Mixed/other	-0.24	-0.54,0.06	0.12
Highest educational qualification	University degree or higher			
	Qualifications below degree	0.18	0.07,0.29	0.001
	No qualifications	0.04	-0.10,0.18	0.59
Household income tertile	Lowest			
	Middle	0.12	0.00,0.25	0.05
	Highest	0.10	-0.03,0.22	0.12
Subjective income	Low			
	Middle	0.08	-0.01,0.18	0.09
	High	0.13	-0.08,0.34	0.22
Occupational social class: NS-SEC	Higher managerial, professional, administrative			
	Intermediate	0.16	0.02,0.31	0.03
	Small employers/own account	0.15	-0.01,0.32	0.07
	Lower supervisory and technical	0.15	-0.01,0.31	0.06
	Semi-routine and routine	-0.02	-0.13,0.09	0.68
	Unclassifiable	-0.16	-0.45,0.13	0.29
Employment status	Employed			
	Unemployed	-0.02	-0.25,0.22	0.87
	Permanently sick/disabled	-0.12	-0.36,0.11	0.31
	Other	0.01	-0.12,0.13	0.93
BMI, categorized†	18.5-24.9	Ref		
	25.0-29.9	-0.19	-0.29,-0.09	<0.001
	30.0-34.9	-0.38	-0.52,-0.23	<0.001

	35+	-0.86	-1.04,-0.67	<0.001
	<18.5	0.09	-0.24,0.42	0.59
Perception of own weight	About the right weight			
	Underweight or very underweight	-0.16	-0.34,0.02	0.08
	A bit overweight	-0.25	-0.34,-0.15	<0.001
	Very overweight	-0.83	-1.00,-0.65	<0.001
Long-term illness or disability (standardized)	No	Ref		
	Yes	-0.26	-0.36,-0.17	<0.001

*all models included gender and age categories. †Based on a separate imputation model in which BMI was imputed in categories.

Table S4a. Age differences in weight-stigmatizing attitudes

**Age group: 30-39**	**Coeff**	**CI**	**p**
Base model	-0.02	-0.19,0.15	0.83
+ own BMI	0.01	-0.15,0.18	0.88
+ view of own weight	0.01	-0.16,0.17	0.95
+ happiness with own weight	0.00	-0.17,0.17	0.98
+ longterm illness/disability	-0.01	-0.18,0.16	0.90
+ causes of obesity: choice vs biology	-0.01	-0.17,0.16	0.91
+ causes of obesity: structural factors	-0.02	-0.19,0.15	0.80
+ health consequences of obesity	-0.01	-0.18,0.16	0.88
+ left-right values	-0.03	-0.2,0.14	0.72
+ libertarian-authoritarian values	-0.05	-0.22,0.11	0.53
+ welfare-stigmatizing attitudes	-0.01	-0.17,0.15	0.87
Full adjustment	0.01	-0.14,0.16	0.91
**Age group: 40-49**	**Coeff**	**CI**	**p**
Base model	-0.05	-0.21,0.12	0.58
+ own BMI	0.03	-0.13,0.19	0.67
+ view of own weight	-0.01	-0.17,0.16	0.94
+ happiness with own weight	-0.03	-0.2,0.13	0.69
+ longterm illness/disability	-0.02	-0.18,0.14	0.81
+ causes of obesity: choice vs biology	-0.07	-0.23,0.09	0.39
+ causes of obesity: structural factors	-0.05	-0.22,0.11	0.51
+ health consequences of obesity	-0.05	-0.21,0.12	0.58
+ left-right values	-0.06	-0.22,0.11	0.49
+ libertarian-authoritarian values	-0.1	-0.26,0.06	0.24
+ welfare-stigmatizing attitudes	-0.04	-0.20,0.11	0.60
Full adjustment	-0.01	-0.16,0.13	0.86
**Age group: 50-59**	**Coeff**	**CI**	**p**
Base model	-0.27	-0.44,-0.11	0.001
+ own BMI	-0.18	-0.34,-0.01	0.03
+ view of own weight	-0.19	-0.35,-0.03	0.02
+ happiness with own weight	-0.26	-0.42,-0.10	0.002
+ longterm illness/disability	-0.24	-0.4,-0.08	0.004
+ causes of obesity: choice vs biology	-0.31	-0.47,-0.16	<0.001
+ causes of obesity: structural factors	-0.28	-0.44,-0.12	0.001
+ health consequences of obesity	-0.27	-0.43,-0.11	0.001
+ left-right values	-0.28	-0.44,-0.12	0.001
+ libertarian-authoritarian values	-0.30	-0.46,-0.15	<0.001
+ welfare-stigmatizing attitudes	-0.21	-0.37,-0.06	0.007
Full adjustment	-0.15	-0.30,0.00	0.05

*All models included age and gender

Table S4b. Age differences in weight-stigmatizing attitudes

Age group: 60-69	Coeff	CI	p
Base model*	-0.14	-0.3,0.02	0.08
+ own BMI	-0.04	-0.2,0.11	0.60
+ view of own weight	-0.07	-0.23,0.08	0.35
+ happiness with own weight	-0.14	-0.29,0.02	0.09
+ longterm illness/disability	-0.07	-0.23,0.09	0.40
+ causes of obesity: choice vs biology	-0.17	-0.32,-0.02	0.03
+ causes of obesity: structural factors	-0.15	-0.31,0.01	0.06
+ health consequences of obesity	-0.12	-0.28,0.04	0.15
+ left-right values	-0.14	-0.29,0.02	0.09
+ libertarian-authoritarian values	-0.23	-0.38,-0.07	0.004
+ welfare-stigmatizing attitudes	-0.15	-0.29,0.00	0.05
Full adjustment	-0.08	-0.22,0.07	0.30
**Age group: 70-79**	**Coeff**	**CI**	**p**
Base model	0.08	-0.11,0.26	0.41
+ own BMI	0.15	-0.03,0.33	0.11
+ view of own weight	0.12	-0.06,0.30	0.20
+ happiness with own weight	0.07	-0.12,0.25	0.49
+ longterm illness/disability	0.18	-0.01,0.37	0.06
+ causes of obesity: choice vs biology	0.07	-0.11,0.25	0.43
+ causes of obesity: structural factors	0.07	-0.11,0.25	0.45
+ health consequences of obesity	0.11	-0.07,0.30	0.24
+ left-right values	0.08	-0.11,0.26	0.41
+ libertarian-authoritarian values	-0.07	-0.25,0.12	0.49
+ welfare-stigmatizing attitudes	0.00	-0.17,0.18	0.99
Full adjustment	0.08	-0.09,0.26	0.37
**Age group: 80+**	**Coeff**	**CI**	**p**
Base model	0.11	-0.1,0.32	0.31
+ own BMI	0.12	-0.08,0.33	0.24
+ view of own weight	0.08	-0.12,0.29	0.43
+ happiness with own weight	0.07	-0.14,0.28	0.51
+ longterm illness/disability	0.23	0.02,0.44	0.04
+ causes of obesity: choice vs biology	0.11	-0.09,0.31	0.28
+ causes of obesity: structural factors	0.11	-0.1,0.32	0.32
+ health consequences of obesity	0.16	-0.06,0.37	0.15
+ left-right values	0.09	-0.12,0.3	0.40
+ libertarian-authoritarian values	0.00	-0.21,0.21	1.00
+ welfare-stigmatizing attitudes	0.03	-0.17,0.22	0.80
Full adjustment	0.08	-0.11,0.28	0.41

*All models included gender

Table S5. Gender differences in weight-stigmatizing attitudes*

**Women vs men**	**Coeff**	**CI**	**p**
Base model*	-0.23	-0.32,-0.14	<0.001
+ own BMI	-0.25	-0.33,-0.16	<0.001
+ view of own weight	-0.20	-0.29,-0.12	<0.001
+ happiness with own weight	-0.20	-0.29,-0.12	<0.001
+ longterm illness/disability	-0.21	-0.3,-0.13	<0.001
+ causes of obesity: choice vs biology	-0.24	-0.33,-0.16	<0.001
+ causes of obesity: structural factors	-0.22	-0.3,-0.13	<0.001
+ health consequences of obesity	-0.23	-0.32,-0.14	<0.001
+ left-right values	-0.24	-0.33,-0.15	<0.001
+ libertarian-authoritarian values	-0.24	-0.32,-0.15	<0.001
+ welfare-stigmatizing attitudes	-0.22	-0.3,-0.13	<0.001
Full adjustment	-0.24	-0.32,-0.16	<0.001

*All models included age

Table S6. Education differences in weight-stigmatizing attitudes

**Qualifications below degree**	**Coeff**	**CI**	**p**
Base model	0.18	0.07,0.29	0.001
+ own BMI	0.24	0.14,0.35	<0.001
+ view of own weight	0.21	0.10,0.32	<0.001
+ happiness with own weight	0.19	0.08,0.30	0.001
+ longterm illness/disability	0.19	0.08,0.30	<0.001
+ causes of obesity: choice vs biology	0.21	0.11,0.32	<0.001
+ causes of obesity: structural factors	0.19	0.08,0.30	0.001
+ health consequences of obesity	0.19	0.08,0.30	0.001
+ left-right values	0.18	0.07,0.29	0.001
+ libertarian-authoritarian values	0.00	-0.11,0.11	0.99
+ welfare-stigmatizing attitudes	0.04	-0.07,0.14	0.50
Full adjustment	0.08	-0.02,0.18	0.127
**No qualifications**	**Coeff**	**CI**	**p**
Base model	0.04	-0.1,0.18	0.59
+ own BMI	0.13	-0.01,0.27	0.08
+ view of own weight	0.06	-0.08,0.20	0.40
+ happiness with own weight	0.05	-0.1,0.19	0.53
+ longterm illness/disability	0.08	-0.06,0.22	0.27
+ causes of obesity: choice vs biology	0.12	-0.02,0.26	0.08
+ causes of obesity: structural factors	0.08	-0.06,0.23	0.25
+ health consequences of obesity	0.07	-0.08,0.21	0.35
+ left-right values	0.08	-0.07,0.22	0.30
+ libertarian-authoritarian values	-0.19	-0.33,-0.04	0.01
+ welfare-stigmatizing attitudes	-0.13	-0.27,0.00	0.06
Full adjustment	0.00	-0.14,0.13	0.96

*All models included age and gender

Table S7. Objective household income differences in weight-stigmatizing attitudes

**Middle tertile**	**Coeff**	**CI**	**p**
Base model	0.12	0.00,0.25	0.05
+ own BMI	0.10	-0.02,0.22	0.10
+ view of own weight	0.10	-0.01,0.22	0.08
+ happiness with own weight	0.12	0.00,0.24	0.05
+ longterm illness/disability	0.09	-0.04,0.21	0.16
+ causes of obesity: choice vs biology	0.08	-0.04,0.19	0.19
+ causes of obesity: structural factors	0.10	-0.02,0.23	0.10
+ health consequences of obesity	0.12	-0.01,0.24	0.06
+ left-right values	0.10	-0.02,0.22	0.12
+ libertarian-authoritarian values	0.14	0.03,0.26	0.02
+ welfare-stigmatizing attitudes	0.09	-0.02,0.21	0.11
Full adjustment	0.01	-0.1,0.12	0.86
**Highest tertile**	**Coeff**	**CI**	**p**
Base model	0.10	-0.03,0.22	0.12
+ own BMI	0.06	-0.06,0.18	0.31
+ view of own weight	0.09	-0.03,0.21	0.16
+ happiness with own weight	0.11	-0.02,0.23	0.09
+ longterm illness/disability	0.04	-0.08,0.17	0.52
+ causes of obesity: choice vs biology	0.02	-0.1,0.15	0.70
+ causes of obesity: structural factors	0.07	-0.06,0.19	0.30
+ health consequences of obesity	0.08	-0.04,0.21	0.20
+ left-right values	0.05	-0.08,0.18	0.46
+ libertarian-authoritarian values	0.19	0.07,0.31	0.00
+ welfare-stigmatizing attitudes	0.08	-0.04,0.20	0.18
Full adjustment	-0.03	-0.14,0.09	0.66

*All models included age and gender

Table S8. Subjective income differences in weight-stigmatizing attitudes

**Middle**	**Coeff**	**CI**	**p**
Base model	0.08	-0.01,0.18	0.09
+ own BMI	0.05	-0.05,0.14	0.33
+ view of own weight	0.07	-0.03,0.16	0.16
+ happiness with own weight	0.08	-0.02,0.17	0.12
+ longterm illness/disability	0.05	-0.05,0.14	0.34
+ causes of obesity: choice vs biology	0.03	-0.06,0.12	0.53
+ causes of obesity: structural factors	0.07	-0.03,0.17	0.17
+ health consequences of obesity	0.07	-0.02,0.17	0.13
+ left-right values	0.06	-0.04,0.15	0.27
+ libertarian-authoritarian values	0.14	0.04,0.23	0.00
+ welfare-stigmatizing attitudes	0.06	-0.03,0.15	0.17
Full adjustment	-0.02	-0.11,0.07	0.68
**High**	**Coeff**	**CI**	**p**
Base model	0.13	-0.08,0.34	0.22
+ own BMI	0.04	-0.16,0.25	0.67
+ view of own weight	0.09	-0.11,0.29	0.37
+ happiness with own weight	0.11	-0.1,0.31	0.30
+ longterm illness/disability	0.09	-0.12,0.29	0.40
+ causes of obesity: choice vs biology	0.00	-0.2,0.2	0.98
+ causes of obesity: structural factors	0.09	-0.12,0.3	0.39
+ health consequences of obesity	0.11	-0.09,0.32	0.28
+ left-right values	0.08	-0.13,0.29	0.46
+ libertarian-authoritarian values	0.34	0.13,0.54	0.00
+ welfare-stigmatizing attitudes	0.21	0.02,0.41	0.03
Full adjustment	0.07	-0.12,0.25	0.49

*All models included age and gender

Table S9. Associations from ordered logistic regression: relative's partner's weight would affect how I felt about the relationship*

**Continuous variables**		**OR**	**CI**	**p**
Own BMI (per 5kg/m^2)		0.66	0.59,0.73	<0.001
Happiness with own weight (per unit, range 1-5)		1.20	1.08,1.33	<0.001
Causes of obesity: choice vs biology (standardized)		0.82	0.75,0.9	<0.001
Causes of obesity: structural factors (standardized)		0.89	0.81,0.97	0.01
Health consequences of obesity (standardized)		1.09	0.99,1.2	0.07
Left-right values (standardized)		1.05	0.95,1.15	0.36
Libertarian-authoritarian values (standardized)		0.91	0.83,1.00	0.05
Welfare-stigmatizing attitudes (standardized)		1.03	0.94,1.13	0.56
**Categorical variables**		**Coeff**	**CI**	**p**
Age group	18-29	Ref		
	30-39	0.82	0.57,1.19	0.30
	49-49	1.13	0.79,1.61	0.51
	50-59	1.20	0.85,1.7	0.30
	60-69	1.29	0.92,1.81	0.14
	70-79	1.77	1.23,2.56	0.002
	80+	1.41	0.92,2.17	0.11
Gender	Men	Ref		
	Women	0.80	0.67,0.97	0.02
Ethnicity	White	Ref		
	Black	0.93	0.52,1.66	0.80
	Asian	1.43	0.93,2.19	0.10
	Mixed/other	1.49	0.78,2.83	0.23
Highest educational qualification	University degree or higher	Ref		
	Qualifications below degree	0.63	0.5,0.78	<0.001
	No qualifications	0.39	0.29,0.53	<0.001
Household income tertile	Lowest	Ref		
	Middle	1.35	1.05,1.74	0.02
	Highest	1.33	1.01,1.75	0.04
Subjective income	Low	Ref		
	Middle	1.45	1.19,1.76	<0.001
	High	3.02	1.96,4.65	<0.001
Occupational social class: NS-SEC	Higher managerial, professional, administrative	Ref		
	Intermediate	0.91	0.67,1.23	0.54
	Small employers/own account	1.04	0.74,1.47	0.81
	Lower supervisory and technical	0.59	0.42,0.84	0.003
	Semi-routine and routine	0.65	0.51,0.82	<0.001
	Unclassifiable	1.14	0.65,2	0.66
Employment status	Employed	Ref		
	Unemployed	0.77	0.47,1.27	0.31
	Permanently sick/disabled	0.62	0.37,1.05	0.07
	Other	1.11	0.86,1.45	0.42
Perception of own weight	About the right weight	Ref		
	Underweight or very underweight	0.85	0.58,1.24	0.39

	A bit overweight	0.59	0.48,0.73	<0.001
	Very overweight	0.29	0.18,0.45	<0.001
Long-term illness or disability (standardized)	No	Ref		
	Yes	0.79	0.65,0.97	0.02

*All models included gender, age and age squared

Table S10. Associations from linear regression: individual items in the index of weight-stigmatizing attitudes*

		Lazy†			Lose†			NHS†			Care†		
**Continuous variables**		**Coeff**	**CI**	**p**	**Coeff**	**CI**	**p**	**Coeff**	**CI**	**p**	**Coeff**	**CI**	**p**
Own BMI (per 5kg/m^2)		-0.16	-0.2,-0.12	<0.001	-0.11	-0.16,-0.07	<0.001	-0.18	-0.23,-0.14	<0.001	-0.15	-0.19,-0.10	<0.001
Happiness with own weight (per unit, range 1-5)		0.08	0.03,0.12	0.001	0.11	0.06,0.16	<0.001	0.09	0.04,0.13	<0.001	0.08	0.03,0.13	0.001
Causes of obesity: choice vs biology†		-0.20	-0.25,-0.15	<0.001	-0.21	-0.25,-0.16	<0.001	-0.18	-0.22,-0.14	<0.001	-0.15	-0.19,-0.1	<0.001
Causes of obesity: structural factors†		0.00	-0.05,0.05	0.97	-0.04	-0.08,0.01	0.11	-0.10	-0.15,-0.06	<0.001	-0.07	-0.12,-0.02	0.005
Health consequences of obesity†		0.03	-0.02,0.08	0.19	<0.001	-0.04,0.05	0.838	0.08	0.04,0.13	<0.001	0.03	-0.01,0.08	0.17
Left-right values†		0.02	-0.03,0.07	0.47	0.03	-0.02,0.07	0.274	0.13	0.08,0.18	<0.001	0.08	0.03,0.13	0.001
Libertarian-authoritarian values†		0.21	0.17,0.26	<0.001	0.23	0.19,0.28	<0.001	0.14	0.09,0.19	<0.001	0.06	0.01,0.10	0.02
Welfare-stigmatizing attitudes†		0.3	0.25,0.34	<0.001	0.31	0.27,0.36	<0.001	0.23	0.18,0.27	<0.001	0.12	0.07,0.16	<0.001
**Categorical variables**		**Coeff**	**CI**	**p**	**Coeff**	**CI**	**p**	**Coeff**	**CI**	**p**	**Coeff**	**CI**	**p**
Age group	18-29												
	30-39	-0.26	-0.43,-0.09	0.002	-0.08	-0.26,0.1	0.39	0.14	-0.04,0.31	0.12	0.16	-0.02,0.34	0.08
	49-49	-0.30	-0.46,-0.13	<0.001	-0.21	-0.38,-0.04	0.015	0.19	0.03,0.36	0.02	0.20	0.03,0.37	0.02
	50-59	-0.58	-0.75,-0.42	<0.001	-0.33	-0.50,-0.16	<0.001	0.11	-0.06,0.28	0.19	0.05	-0.11,0.22	0.54
	60-69	-0.58	-0.74,-0.42	<0.001	-0.24	-0.41,-0.07	0.005	0.25	0.09,0.41	0.003	0.21	0.04,0.37	0.014
	70-79	-0.45	-0.63,-0.27	<0.001	-0.06	-0.25,0.12	0.504	0.42	0.23,0.62	<0.001	0.32	0.13,0.51	0.001
	80+	-0.44	-0.66,-0.22	<0.001	0.01	-0.2,0.23	0.923	0.41	0.18,0.63	<0.001	0.35	0.13,0.57	0.002
Gender	Men												
	Women	-0.19	-0.28,-0.1	<0.001	-0.26	-0.35,-0.17	<0.001	0.00	-0.09,0.09	0.95	-0.20	-0.29,-0.10	<0.001
Ethnicity	White												
	Black	-0.19	-0.48,0.1	0.20	0.25	-0.04,0.54	0.09	-0.15	-0.44,0.14	0.31	-0.16	-0.45,0.14	0.31
	Asian	0.28	0.08,0.49	0.006	0.28	0.08,0.48	0.01	-0.41	-0.61,-0.20	<0.001	-0.08	-0.29,0.13	0.46
	Mixed/other	-0.05	-0.36,0.26	0.755	-0.05	-0.37,0.26	0.75	-0.32	-0.62,-0.01	0.04	-0.24	-0.56,0.08	0.14
Highest educational qualification	University degree												
	Qualifications below degree	0.11	0.00,0.21	0.05	0.16	0.05,0.27	0.005	0.19	0.07,0.30	0.001	0.04	-0.07,0.16	0.46
	No qualifications	0.19	0.05,0.33	0.01	0.21	0.07,0.35	0.004	-0.15	-0.31,0.00	0.05	-0.14	-0.30,0.01	0.07

Household income tertile	Lowest												
	Middle	-0.02	-0.15,0.1	0.695	0.09	-0.02,0.21	0.12	0.21	0.09,0.33	0.001	0.06	-0.06,0.18	0.36
	Highest	-0.02	-0.15,0.1	0.701	0.07	-0.05,0.19	0.26	0.18	0.06,0.31	0.005	0.04	-0.09,0.17	0.56
Subjective income	Low												
	Middle	-0.01	-0.11,0.08	0.806	0.04	-0.06,0.14	0.40	0.14	0.04,0.23	0.005	0.07	-0.04,0.17	0.20
	High	-0.01	-0.22,0.2	0.941	0.01	-0.2,0.22	0.93	0.24	0.02,0.45	0.03	0.12	-0.10,0.33	0.28
Occupational social class: NS-SEC	Higher managerial, professional, administrative												
	Intermediate	0.08	-0.06,0.23	0.266	0.12	-0.03,0.27	0.11	0.14	-0.01,0.29	0.06	0.10	-0.05,0.25	0.19
	Small employers/ own account	0.21	0.04,0.39	0.016	0.13	-0.04,0.3	0.14	0.12	-0.06,0.29	0.19	-0.04	-0.21,0.13	0.62
	Lower supervisory and technical	0.05	-0.12,0.21	0.587	0.20	0.03,0.36	0.02	0.07	-0.09,0.23	0.37	0.10	-0.06,0.27	0.23
	Semi-routine and routine	0.13	0.02,0.24	0.018	0.07	-0.04,0.18	0.21	-0.14	-0.26,-0.03	0.014	-0.13	-0.25,-0.01	0.03
	Unclassifiable	0.14	-0.16,0.44	0.355	0.09	-0.22,0.39	0.57	-0.44	-0.71,-0.16	0.002	-0.23	-0.53,0.07	0.14
Employment status	Employed												
	Unemployed	0.08	-0.15,0.3	0.515	-0.02	-0.25,0.21	0.89	-0.07	-0.3,0.17	0.57	-0.04	-0.28,0.19	0.70
	Permanently sick/disabled	0.08	-0.17,0.33	0.514	0.09	-0.15,0.34	0.45	-0.32	-0.56,-0.08	0.009	-0.20	-0.45,0.05	0.12
	Other	0.04	-0.09,0.16	0.558	0.07	-0.06,0.2	0.27	-0.06	-0.19,0.06	0.33	-0.03	-0.16,0.10	0.65
Perception of own weight	About the right weight												
	Underweight or very underweight	-0.1	-0.29,0.1	0.332	-0.05	-0.23,0.13	0.59	-0.21	-0.4,-0.02	0.034	-0.09	-0.28,0.1	0.36
	A bit overweight	-0.17	-0.27,-0.07	0.001	-0.17	-0.26,-0.07	0.001	-0.2	-0.3,-0.1	<0.001	-0.16	-0.26,-0.06	0.001
	Very overweight	-0.62	-0.79,-0.44	<0.001	-0.55	-0.73,-0.37	<0.001	-0.58	-0.77,-0.4	<0.001	-0.54	-0.72,-0.36	<0.001
Long-term illness or disability	No												
	Yes	-0.16	-0.25,-0.06	0.002	-0.19	-0.29,-0.09	<0.001	-0.19	-0.28,-0.09	<0.001	-0.19	-0.29,-0.1	<0.001

***All models included age and gender. †Variable was standardized.**
